# The Key Role of Fatty Acid Synthase in Lipid Metabolism and Metamorphic Development in a Destructive Insect Pest, *Spodoptera litura* (Lepidoptera: Noctuidae)

**DOI:** 10.3390/ijms23169064

**Published:** 2022-08-13

**Authors:** Yan Song, Fengming Gu, Zhixiang Liu, Zongnan Li, Fu’an Wu, Sheng Sheng

**Affiliations:** 1 School of Biotechnology, Jiangsu University of Science and Technology, Zhenjiang 212100, China; 2 The Key Laboratory of Silkworm and Mulberry Genetic Improvement, Ministry of Agriculture and Rural Affairs, Sericultural Research Institute, Chinese Academy of Agricultural Science, Zhenjiang 212100, China

**Keywords:** fatty acid synthase (FAS), *Spodoptera litura*, lipid metabolism, metamorphosis, RNA interference

## Abstract

Fatty acid synthase (FAS) is a key enzyme in the lipid synthesis pathway, however, its roles in insects remain largely unknown. Here, we firstly identified two *FAS* genes from the transcriptome dataset of the general cutworm *Spodoptera litura*, which is a destructive insect pest of many crops. Both *SlFAS1* and *SlFAS2* were highly expressed in third instar larvae and in their fat bodies. Then, we successfully silenced *SlFAS1* in third instar larvae and the content of α-linolenic acid and triglyceride was significantly decreased. Besides that, the effect of FAS on the metamorphic development in *S. litura* was evaluated. The results indicate that after silencing *SlFAS1*, the survival rates of *S. litura* larvae decreased significantly compared to the control groups. Silencing *SlFAS1* in fifth instar larvae resulted in more malformed pupae and adults, and the emergence rates were significantly reduced. Furthermore, the ecdysone content in the haemolymph of fifth instar larvae was significantly decreased after silencing *SlFAS1*. In addition, knocking down *SlFAS1* significantly alters the expression of other key genes in the lipogenesis pathway, implying that FAS has an impact on the lipogenesis pathway. The present study deepens the understanding of FAS in insects and provides novel potential targets for managing insect pests.

## 1. Introduction

Lipids are essential in organisms and play multiple roles such as energy storage, signal transduction, hormone synthesis, and cell membrane component formation, and they are vital for maintaining normal life activities. Lipid synthesis is a highly complex process catalysed by a battery of enzymes, among which fatty acid synthases (FASs) are key and multifunctional enzymes in fatty acid synthesis and lipid metabolism. There are two types of FASs existing in organisms. Type I FASs are predominantly found in animals and fungi. The type I FASs of fungi are encoded by two genes that assemble an α6β6-heterododecamer [1], but mammalian type I FASs are expressed as a single protein and assemble as a homodimer [2]. In contrast, type Ⅱ FASs are common in bacteria and eukaryotic organelles, found especially in chloroplasts and mitochondria, and are expressed as discrete proteins in the cytosol [3]. The general FAS monomer contains seven functional domains including β-ketoacyl synthase (KS), malonyl/acetyl transferase (MAT), β-hydroxyacyl dehydratase (DH), enoyl reductase (ER), β-ketoacyl reductase (KR), acyl carrier protein (ACP), and thioesterase (TE) [4].

Until now, the identification, as well as the function, of *FAS* genes has only been studied in a narrow range of insect species [5,6]. The first reported insect FAS was purified from the fat body of *Drosophila melanogaster* and there are three *FAS* genes (*FAS^CG3523^*, *FAS^CG3524^*, *FAS^CG17374^*) identified from *D. melanogaster* [7]. *FAS^CG3523^* is expressed in the fat body while *FAS^CG3524^* and *FAS^CG17374^* are expressed in oenocytes. Silencing *FAS^CG3524^* and *FAS^CG17374^* can induce the lethal phenotype [8]. Generally, *FAS* genes are mainly expressed in the fat body and oenocytes, and some others are also expressed in different tissues with different biological functions [9]. In the fat body, FASs are responsible for the biosynthesis and storage of lipids, mainly deposited in the form of triglyceride, and transport it to different tissues in the form of diacylglycerols through the haemolymph [10]. In *Aedes aegypti*, the expression level of *FAS1* was highest in the fat body after 48 h, but highest in the ovary 72 h after blood feeding. Besides that, triglyceride and phospholipid levels of *A. aegypti* adults were significantly reduced after silencing *FAS1* [11].

Triglyceride is the main component of lipids and FAS regulates *de novo* lipogenesis by converting acetyl-CoA to palmitate, further leading to the production and storage of triglyceride [12]. The fatty acid synthesis pathway also involves other enzymes, such as acetyl-CoA carboxylase (ACC), desaturases (Desat), and lipase. Furthermore, considering the importance of triglyceride in insect development, the exact roles of FASs still receive less attention. Previous research revealed that silencing *GpylFAS1* in a mulberry pest, *Glyphodes pyloalis*, significantly reduced the content of α-linolenic acid, resulting in more malformed pupa and lower emergence rates [13]. Studies have shown that two hormones in insects, juvenile hormone and ecdysone, also participate in the regulation of triglyceride metabolism [14]. However, it remains unknown whether FAS regulates the dynamics of juvenile hormone and ecdysone during metamorphic development.

The general cutworm, *Spodoptera litura* (Fabricius) (Lepidoptera: Noctuidae) is a serious worldwide polyphagous pest. It causes heavy damage to dozens of crops, such as potato, tomato, corn, and mulberry trees every year [15]. At present, studies on lipid metabolism in *S. litura* have been reported [16], but there is no reference to the role of FASs in regulating lipid metabolism in *S. litura*. 

In the present study, we obtained two *FAS* genes from the transcriptome of *S. litura* and focused on the role of *SlFASs* in the synthesis of lipids and in the development and metamorphosis of *S. litura* larvae. The present study reveals the molecular mechanism of FAS during the development of *S. litura*, providing a novel potential target for the integrated biological control of pests.

## 2. Results

### 2.1. Gene Identification and Sequence Analysis of FAS Genes in S. litura

**Figure 1:**
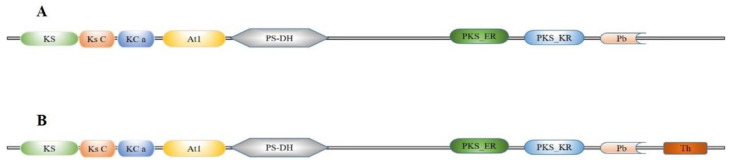
Domain schematic of SlFAS1 (**A**) and SlFAS2 (**B**) in *S. litura*. KS, Ketoacyl-synt (pfam00109); Ks C, Ketoacyl-synt_C (pfam02801); KC a, KAsynt_C_assoc (pfam16197); At1, Acyl_transf_1 (pfam00698); PS-DH (PF14765); PKS_ER (smart00829); PKS_KR (smart00822); Pb, PP-binding (pfam00550); Th, Thioesterase (pfam00975).

**Figure 2:**
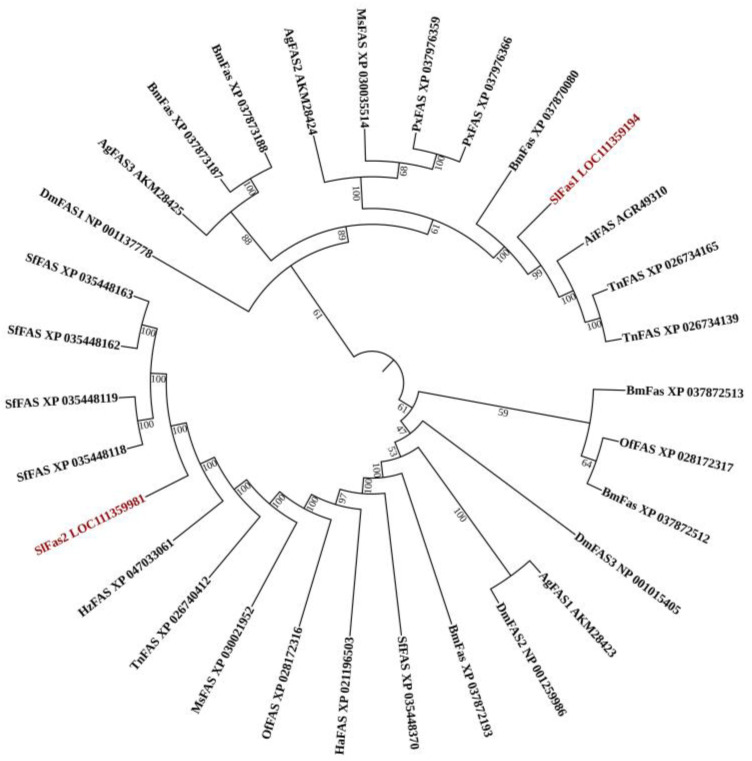
Phylogenetic tree of FAS amino acid sequences in different species. *Spodoptera litura* (Sl), *Bombyx mori* (Bm), *Drosophila melanogaster* (Dm), *Spodoptera frugiperda* (Sf), *Helicoverpa zea* (Hz), *Trichoplusia ni* (Tn), *Ostrinia furnacalis* (Of), *Helicoverpa armigera* (Ha), *Plutella xylostella* (Px), *Agrotis ipsilon* (Ai).

Two *FAS* genes were identified from our previously constructed *S. litura* larvae transcriptome database and named *SlFAS1* (LOC111359194) and *SlFAS2* (LOC111359981). The sequences of SlFAS1 and SlFAS2 contained complete ORFs of 6240 and 7269 bp which encoded 2079 and 2422 amino acid residues, respectively. The protein sequences of SlFAS1 have eight functional domains, including Ketoacyl-synt (ks, pfam00109), Ketoacyl-synt_C (KsC, pfam02801), KAsynt_C_assoc (KCa, pfam16197), Acyl_transf_1 (At1, pfam00698), PS-DH (PF14765), PKS_ER (smart00829), PKS_KR (smart00822), and PP-binding (Pb, pfam00550). However, SlFAS2 contains nine functional domains, with an additional Thioesterase domain (Th, pfam00975), compared to SlFAS1 (Figure 1). Sequence alignment showed 35.63% amino acid identity between SlFAS1 and SlFAS2 (Figure S1). Phylogenetic analysis of FASs revealed that SlFAS1 and FASs in *Agrotis ipsilon* and *Trichoplusia ni* were clustered into a subcluster, and SlFAS2 was clustered with FASs in *Spodoptera frugiperda* into a subbranch (Figure 2).

### 2.2. Expression Patterns of SlFAS1 and SlFAS2

**Figure 3:**
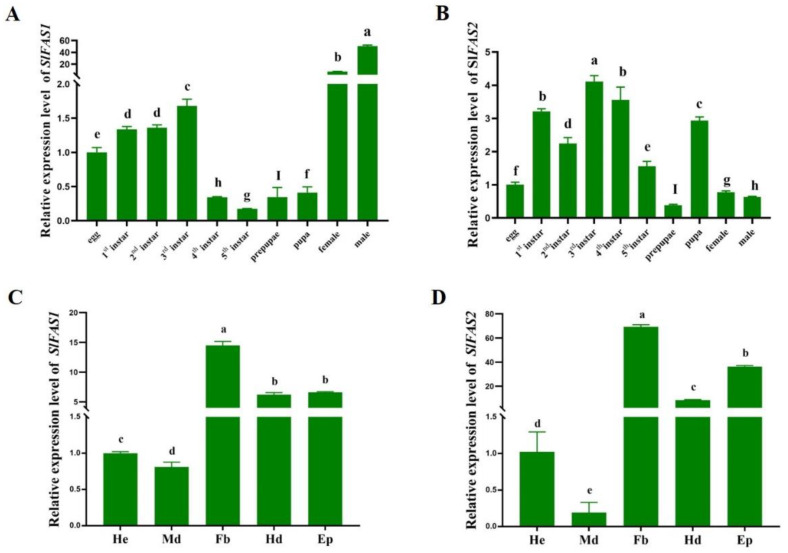
Expression of *SlFAS1* and *SlFAS2* in different development stages and tissues. (**A**) Expression patterns of *SlFAS1* in different development stages. (**B**) Expression patterns of *SlFAS2* in different development stages. (**C**) Expression patterns of *SlFAS1* in different tissues. (**D**) Expression patterns of *SlFAS2* in different tissues. He, haemolymph; Md, midgut; Fb, fat body; Hd, head; Ep, epidermis. Significant differences in expression levels among developmental stages and different tissues were compared using a one-way analysis of variance (ANOVA) with Tukey’s post-hoc test. Different lowercase letters above the bars indicate significant differences (*p* < 0.05).

*SlFAS1* had the highest expression level in adults, and the expression level in males was significantly higher than that in females. In the immature stages, the expression level of *SlFAS1* was higher in third instar larvae (Figure 3A). In contrast, *SlFAS2* had the highest expression level in third instar larvae compared to other stages followed by first and fourth instar larvae (Figure 3B). Besides that, both *SlFAS1* and *SlFAS2* were expressed at the highest levels in the fat body and the lowest in the midgut (Figure 3C,D).

### 2.3. Analysis of the Function of SlFASs Using RNAi

**Figure 4:**
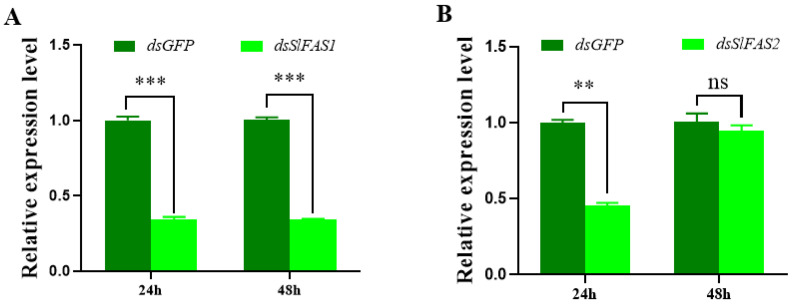
Expression levels of *SlFAS1* and *SlFAS2* after RNAi. (**A**) *SlFAS1* expression levels after *dsSlFAS1* injection. (**B**) *SlFAS2* expression levels after *dsSlFAS2* injection. Significant differences in the expression levels of each target gene were compared using a one-way analysis of variance (ANOVA) followed by Tukey’s post-hoc test. Significant differences are indicated by asterisks (** *p* < 0.01; *** *p* < 0.001; ns: no significant differences).

To further evaluate the function of *SlFASs*, both *SlFAS1* and *SlFAS2* were successfully silenced in third instar *S. litura* larvae, with expression levels decreased significantly 24 h after dsRNA injection (Figure 4A,B). However, silencing *SlFAS2* failed 48 h after dsRNA injection (Figure 4B); therefore, *SlFAS1* was chosen for subsequent functional verification.

**Figure 5:**
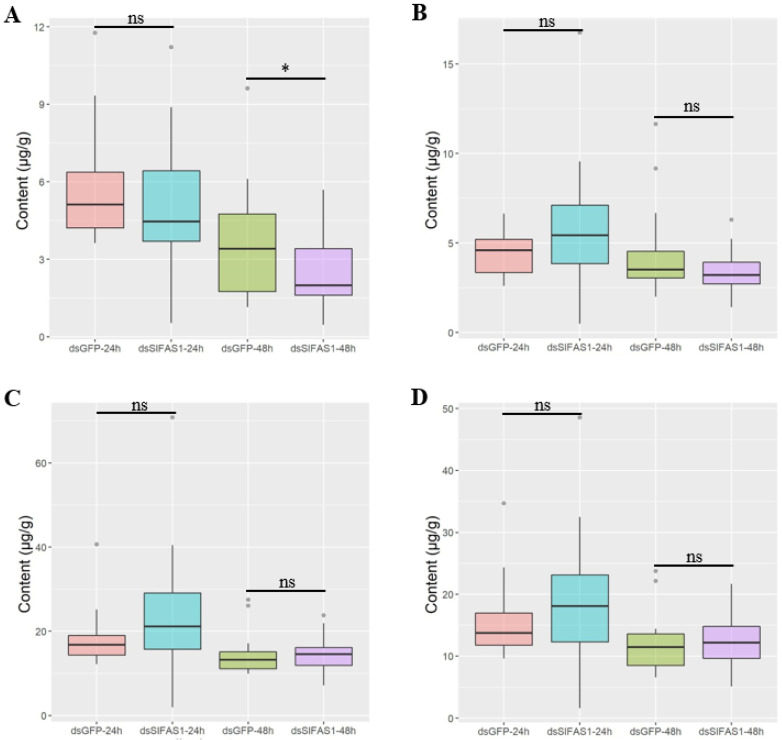
Effect of silencing *SlFAS1* on the fatty acid content in *S. litura* larvae. (**A**) Effect of silencing *SlFAS1* on methyl α-linolenate content. (**B**) Effect of silencing *SlFAS1* on methyl palmitate content. (**C**) Effect of silencing *SlFAS1* on methyl oleate content. (**D**) Effect of silencing *SlFAS1* on methyl linoleate content. Significant differences are indicated by asterisks (* *p* < 0.01; ns: no significant differences; non parametric Mann–Whitney test).

Gas chromatography determination showed that the content of methyl α-linolenate was significantly reduced 48 h after silencing *SlFAS1*, but no obvious change was observed at 24 h compared to the *dsGFP*-injection group (Figure 5A). Moreover, after silencing *SlFAS1*, the content of methyl palmitate, methyl oleate, and methyl linoleate did not change significantly compared to the control cohorts at both 24 and 48 h (Figure 5B–D).

### 2.4. Effect of Silencing SlFAS1 on Lipid Accumulation in S. litura Larvae

**Figure 6:**
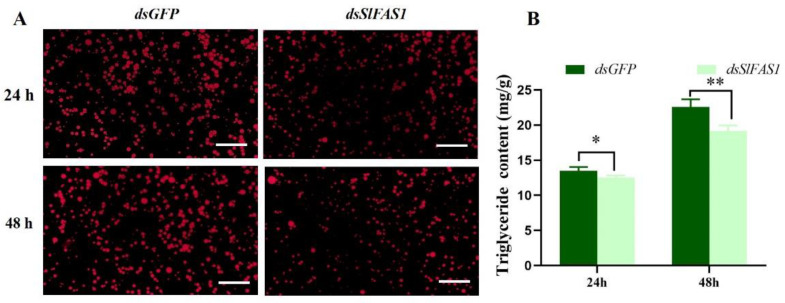
Lipid accumulation in the fat bodies of *S. litura* larvae after *dsSlFAS1*. (**A**) The fat body of *S. litura* was stained with Nile red 24 and 48 h after *dsSlFAS1* injection and examined by laser scanning microscope. Scale bar, 20 µm. (**B**) Triglyceride content in the fat body of *S. litura* after *dsSlFAS1* injection. Differences in the Triglyceride content was compared using a one-way analysis of variance (ANOVA) followed by Tukey’s post-hoc test. Significant differences were indicated by asterisks (* *p* < 0.05; ** *p* < 0.01).

To understand the effect of silencing *SlFAS1* on lipid accumulation in third instar *S. litura* larvae, we detected the triglyceride content after *dsSlFAS1* injection. Nile red staining revealed that the density of lipid droplets was significantly reduced in the fat body at 24 and 48 h compared to the control group after dsRNA injection, respectively (Figure 6A). Besides that, the triglyceride content of the fat body decreased significantly at 24 and 48 h after *dsSlFAS1* injection compared to the *dsGFP* group, respectively (Figure 6B).

### 2.5. Effect of Silencing SlFAS1 on the Development of S. litura Larvae

**Figure 7:**
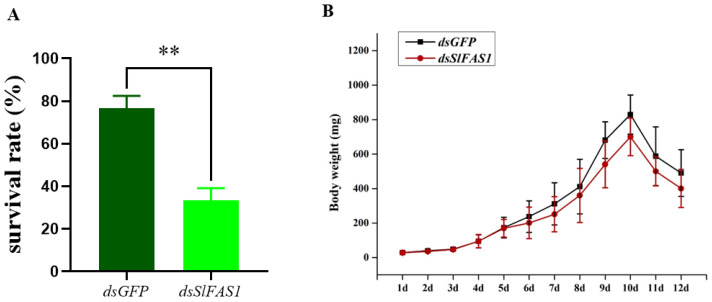
Effect of *SlFAS1* on the development of *S. litura* larvae. (**A**) Survival rate of *S. litura* larvae after injection of *dsSlFAS1* into third instar larvae. (**B**) Growth trajectory of *S. litura* larvae after injection of *dsSlFAS1* into the third instar larvae. Significant differences were compared using a one-way analysis of variance (ANOVA). Significant differences were indicated by asterisks (** *p* < 0.01).

To study the effect of *SlFAS1* on the development of *S. litura* larvae, we analysed survival rates and the growth trajectory of body weight after *dsSlFAS1* injection in third instar larvae. The results showed that the survival rates of *dsSlFAS1*-injection larvae were significantly reduced by approximately 45% compared to the *dsGFP* control (Figure 7A). In addition, compared to the *dsGFP*-injection group, the growth trajectory in terms of body weight at each time point between the *dsSlFAS1*-injection and *dsGFP*-injection groups was not significantly different (Figure 7B).

**Figure 8:**
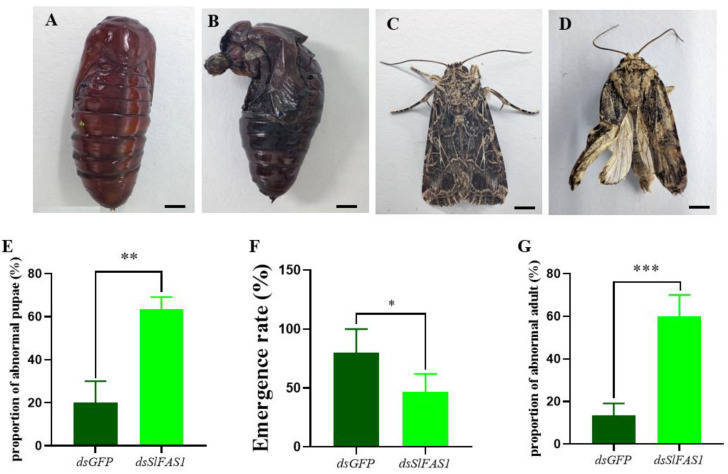
Morphology of pupae and adults after knocking down *SlFAS1* in fifth instar *S. litura*. (**A**) Normal pupae after *dsGFP* injection. (**B**) Abnormal pupae after *dsSlFAS1* injection. (**C**) Normal adults after *dsGFP* injection. (**D**) Abnormal adults after *dsSlFAS1* injection. (**E**) Effect of silencing *SlFAS1* on the proportion of abnormal pupae in *S. litura*. (**F**) Effect of silencing *SlFAS1* on emergence rate in *S. litura*. (**G**) Effect of silencing *SlFAS1* on the proportion of abnormal adults in *S. litura*. Significant differences were compared using a one-way analysis of variance (ANOVA). Significant differences were indicated by asterisks (* *p* < 0.05; ** *p* < 0.01; *** *p* < 0.001); scale bars in A and B: 2 mm; scale bars in C and D: 2 mm.

**Figure 9:**
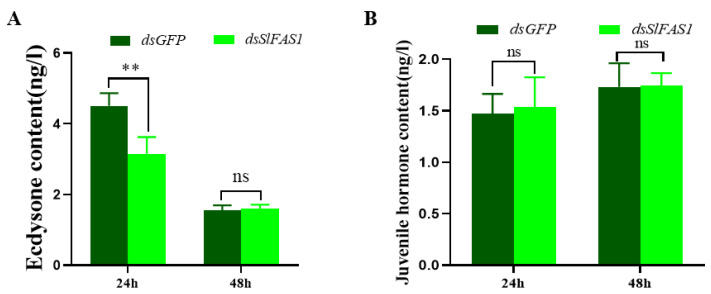
Effect of knocking down *SlFAS1* on the content of juvenile hormone and ecdysone in the haemolymph of fifth instar larvae. (**A**) Effect of silencing *SlFAS1* on ecdysone content in the haemolymph of fifth *S. litura* larvae. (**B**) Effect of silencing *SlFAS1* on juvenile hormone content in the haemolymph of fifth instar *S. litura* larvae. Significant differences were compared using a one-way analysis of variance (ANOVA). Significant differences were indicated by asterisks (** *p* < 0.01; ns, no significant differences).

After the *dsSlFAS1* injection in the fifth instar larvae, the proportion of abnormal pupae was significantly higher compared to the *dsGFP*-injection groups (Figure 8A,B,E). Subsequently, the emergence rate of pupae derived from the *dsSlFAS1*-injected fifth instar larvae was significantly lower than that of the *dsGFP* control group (Figure 8F). Meanwhile, more morphologically abnormal adult moths were observed in the *dsSlFAS1*-injection group than in the *dsGFP*-injection group (Figure 8C,D,G). At the same time, we also determined the content of juvenile hormone and ecdysone in the haemolymph of fifth instar larvae after silencing *SlFAS1*; the results showed that the content of ecdysone decreased significantly 24 h after silencing *SlFAS1*, but no significant change was observed 48 h post knocking down *SlFAS1* (Figure 9A). By contrast, the content of juvenile hormone was changed neither 24 h nor 48 h after silencing *SlFAS1* (Figure 9B).

### 2.6. Effect of Silencing SlFAS1 on the Expression of other Key Genes in the Lipogenesis Pathway

**Figure 10:**
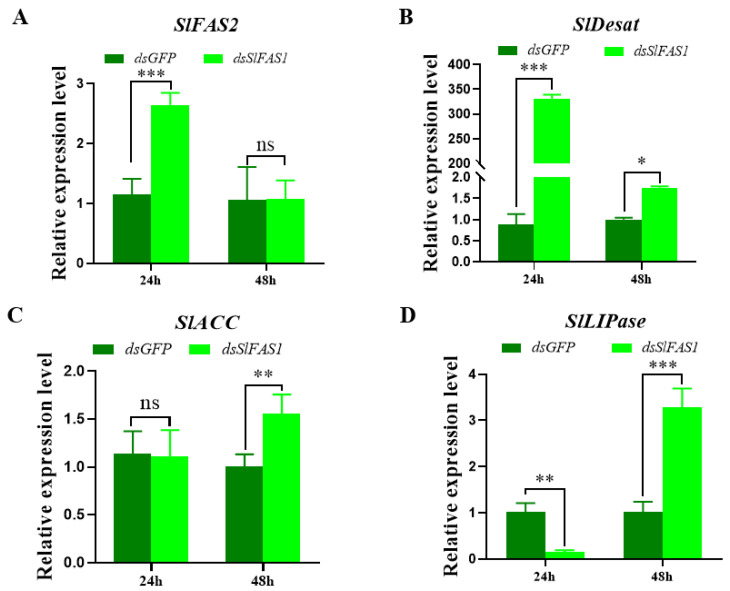
Effect of silencing *SlFAS1* on the expression levels of other key genes in the lipid synthesis pathway in *S. litura* larvae. (**A**) *SlFAS2* expression levels after *dsSlFAS1* injection. (**B**) *SlDesat* expression levels after *dsSlFAS1* injection. (**C**) *SlACC* expression levels after *dsSlFAS1* injection. (**D**) *SlLIPase* expression levels after *dsSlFAS1* injection. One-way analysis of variance (ANOVA) with Tukey’s post hoc test was used to compare significant differences in the expression levels of each target gene. Significant differences were indicated by asterisks (* *p* < 0.05; ** *p* < 0.01; *** *p* < 0.001; ns, no significant differences).

To explore the effect of silencing *SlFAS1* on the lipogenesis pathway, the expression levels of other key genes in the fatty acid synthesis pathway after *dsSlFAS1* injection were validated by qRT-PCR. The expression levels of *SlFAS2* and *SlDesat* were significantly upregulated24 h after *dsSlFAS1* injection, and the expression of *SlDesat* was also increased 48 h after *dsSlFAS1* injection. The expression levels of *SlACC* and *SlLIPase* were significantly upregulated 48 h after *dsSlFAS1* injection; in addition, *SlLIPase* was significantly downregulated 24 h after silencing *SlFAS1* (Figure 10).

## 3. Discussion

Lipid is important for energy homeostasis in insect development, reproduction and hormone synthesis [17]. The synthesis of lipids is an intricate process regulated by multiple enzymes. Among these, FAS is a vital participant mainly contributing to fatty acid synthesis and its functional characterization has been reported in bacteria [18] and fungi [19]. However, the identification and characterization of FAS in insects have still received less attention.

In the present study, we firstly identified two *FAS* genes from our previously constructed transcriptome database. The number of *FAS* genes identified here was similar to that present in other insects, such as *Colaphellus bowringi* (two FAS genes) [20]. The relatively small number suggests the conservative function of FAS. On the other hand, sequence analysis showed that SlFAS1 and SlFAS2 shared only 35.63% amino acid identity with each other, and conserved domain prediction revealed that unlike SlFAS2, SlFAS1 lacks a Thioesterase domain (pfam00975), implying the potential functional divergence of these two genes. 

To explore the function of the *SlFASs*, we analysed their expression patterns in *S. litura* larvae by qRT-PCR. Although *SlFAS1* was expressed highest in adults, it was also expressed higher in third instar larvae, suggesting that *SlFAS1* still plays a role in *S. litura* larvae. Meanwhile, *SlFAS2* was highly expressed in the third instar larvae of *S. litura*. Previous studies have reported this similar development stage-biased expression pattern. The expression levels of *MpulFAS1* and *MpulFAS2* reach a peak at the adult stage but the *MpulFAS3* and *MpulFAS4* expressions peaks at the larval stage in *Meteorus pulchricornis* [21]. The divergence of the *FAS* expression at various different developmental stages indicates that FAS plays distinct functions at different time periods and its role is more complicated. For tissue expression, both *SlFAS1* and *SlFAS2* were highest in fat bodies. This fat body-biased expression is also reported in other insect species and attributed to the main role of the fat body in lipid metabolism [22]. In *Rhodnius prolixus*, *FAS1* is predominantly expressed in the fat body [23]. A high transcript abundance of *FAS2* has been observed in the fat body of diapaused female *Colaphellus bowringi* [20]. Meanwhile, a higher expression of *SlFASs* in other tissues such as the epidermis needs to be noted in future studies for revealing the potential role of FASs in insects.

FAS is a central enzyme in *de novo* lipogenesis, which catalyses the production of C14, C16, and C18 fatty acyl-CoAs, and produces multiple fatty acids [24,25]. Although this catalysis is well summarized in model organisms, there is still a lack of sufficient evidence to support whether FAS undertakes a consistent mission in insects. In the present study, changes in the content of four major fatty acids were examined after silencing *SlFAS1*, and the GC results showed that the content of methyl α-linolenate was decreased significantly only at 48 h after silencing *SlFAS1*. However, no significant changes in the other three fatty acids were observed. This result strongly suggests that *SlFAS1* is involved in α-linolenic acid synthesis in third instar *S. litura* larvae. Wang et al. [13] reported that the content of methyl α-linolenate, as well as methyl palmitate, methyl oleate, and methyl linoleate, is decreased significantly after silencing *GpylFAS1* in the pupae of the lepidopteran pest *Glyphodes pyloalis*. Similarly, knocking down *Bgfas1* significantly reduces the cuticle free fatty acid content in *Blattella germanica* [26,27]. Our results not only show that *SlFAS1* solely affects α-linolenic acid, but also indicate that the synthesis of insect fatty acids may be regulated by diverse *FAS* genes or even other enzymes, and further research is needed to confirm this hypothesis.

The fat body is a crucial biosynthetic and metabolic factory in insects [28], and lipids are mainly stored in the fat body in the form of triglyceride. In the present study, Nile red staining indicated that lipid droplets in the fat body were significantly reduced at 24 and 48 h after *dsSlFAS1* injection compared to the *dsGFP* group. Meanwhile, the triglyceride content was significantly decreased compared to the *dsGFP* control group at 24 and 48 h. These results reveal a close relationship between *SlFAS1* and lipid synthesis in *S. litura* larvae, and this correlation has been reported in a limited number of species. For example, the level of triglyceride was severely decreased after knocking down *FAS1* in the mosquito *Aedes aegypti* [11]. In Serratia-infected *Acyrthosiphon pisum*, the triglyceride content was reduced by feeding with *dsFASN1* compared with the control [29]. Therefore, the present study demonstrates that FAS is not only responsible for fatty acid synthesis, but also affects the lipid dynamic in insects.

It is well accepted that lipid accumulation affects insect growth and development [30]. In the present study, our results show that body weight in each developmental time point was not significantly different between the *dsSlFAS1*-injection larvae and *dsGFP*-injection cohorts. However, the survival rates of the *dsSlFAS1*-injection third *S. litura* larvae were reduced sharply compared to the *dsGFP*-injection group. In the model organism *C. elegans*, silencing *FASN-1* at the larval stage ceases normal development and juvenile nematodes are not able to reach adulthood successfully [31]. In a recent study, Yang et al. [32] reported that the injection of *dsLmFAS1* and *dsLmFAS3* caused about 80% mortality in *Locusta migratoria*. Therefore, FAS may be essential for insect development.

For insects, pupation and emergence are key scenarios of metamorphosis [33,34]. Knocking down *GpylFAS1* or *GpylDesat5*, which are essential for metamorphosis in *G. pyloalis*, leads to more abnormal pupae and adults, and lower emergence rates [13]. Analogously, our results showed that silencing *SlFAS1* can induce many more abnormal pupae and adults, and the emergence rates were decreased significantly. Similarly, knocking down *LdJHAMT*, which is an essential metabolism-related enzyme, causes pupation failure and lower emergence rates in the potato beetle *Leptinotarsa decemlineata* [35]. Furthermore, it is well demonstrated that the hormones in insect larvae significantly shape the process of metamorphosis, and this regulation can be easily measured by monitoring the content of hormones or the expression of hormone genes. Silencing *juvenile hormone acid methyltransferase* (*JHAMT*) in the beetle *Tribolium castaneum* reduces juvenile hormone levels, resulting in the deceleration of lipid catabolism [36]. In the mosquito *A. aegypti*, 20-hydroxyecdysone (20E) can induce the expression of the transcription factor hepatocyte nuclear factor 4 (HNF4), which accelerates β-oxidation and produces the substances and energy required for oocyte development [37]. Interestingly, our results showed that the content of juvenile hormone in the haemolymph of fifth instar *S. litura* was not significantly changed after *SlFAS1* injection, while the content of ecdysone was significantly decreased at 24 h, suggesting a positive linkage between *SlFAS* and the ecdysone. Tan et al. [20] reported that the transcription of *FAS2* is suppressed by the juvenile hormone and the juvenile hormone receptor methoprene-tolerant. It can be speculated that FAS can regulate the ecdysone metabolism and eventually affect insect metamorphosis.

To further explore the effect of silencing *SlFAS1* on other genes in the lipid synthesis pathway, we detected alterations in the expression of *SlFAS2*, *SlDesat*, *SlACC*, and *SlLIPase* by qRT-PCR. Our results suggest that the expression of these genes changes to varying degrees after knocking down *SlFAS1*, indicating that these genes may be connected with *SlFAS1* to jointly regulate the synthesis of lipids in the body. Yang et al. [38] revealed that the glucose-6-phosphate (G6P) metabolism of the host *D. melanogaster* parasitized by *Pachycrepoideus vindemmiae* is affected by *PvG6PDH*; the expression levels of the host G6P-metabolism-related genes changed at different time points after the host was injected with *PvG6PDH*, suggesting the expression of nutrition–related genes can be affected by other genes. In a recent study, Wang et al. [13] also reported that silencing *GpylFAS1* affected the expressions of the other fatty acid synthesis-related genes in *G. pyloalis*. Taken together, it can be concluded that lipid metabolism-related genes can be regulated by FAS and future studies are required to reveal the detailed molecular mechanism of this regulation. 

## 4. Materials and Methods

### 4.1. Insects

The *S. litura* larvae were collected from the mulberry fields at the campus of Jiangsu University of Science and Technology, Zhenjiang City, Jiangsu Province, China, and reared in the insectary [26 ± 2 °C, 60–80% relative humidity, and photoperiod of 14:10 (L:D) h]. The adult moths were fed with 10% (*w*/*w*) honey solution and provided with strips of paper as the substrate for egg deposition in organza-covered cages (23 cm × 23 cm × 21 cm). After hatching, the newly hatched *S. litura* larvae were fed with artificial diets daily under the same conditions.

### 4.2. Bioinformatics Analysis of FAS Genes in S. litura

The sequences of FASs were identified from the previously constructed *S. litura* transcriptome database (BioProject Acc. in NCBI: PRJNA810583). An open reading frame (ORF) finder (https://www.ncbi.nlm.nih.gov/orffinder/) (accessed on 21 February 2021) was used to predict the ORFs of putative *SlFAS1* and *SlFAS2* genes. The NCBI Conserved Domain Search website (https://www.ncbi.nlm.nih.gov/Structure/cdd/wrpsb.cgi) (accessed on 21 February 2021) was used to predict the protein functional domains. The Simple Modular Architecture Research Tool (SMART) online software (Version: 3.3.2, EMBL, Heidelberg, Germany) (http://smart.embl-heidelberg.de/) (accessed on 21 February 2021) was used to predict the conserved motifs. DNAMAN 8.0 (Lynnon Corporation, Quebec City, QC, Canada) was used for multiple alignments of various protein sequences. Phylogenetic analysis was conducted using the neighbour-joining method by Molecular Evolutionary Genetic Analysis 6.0 (MEGA 6.0, Mega Limited, Auckland, New Zealand) software with 1000 bootstrap replications. The Interactive Tree Of Life (iTOL) online tool (https://itol.embl.de) (accessed on 21 February 2021) was used to generate the circular phylogenetic tree. SlFASs homologous amino acid sequences of *Homo sapiens*, *Spodoptera litura*, *Bombyx mori*, *Drosophila melanogaster*, *Spodoptera frugiperda*, *Helicoverpa zea*, *Trichoplusia ni*, *Ostrinia furnacalis*, *Helicoverpa armigera*, *Plutella xylostella*, *Agrotis ipsilon* were downloaded from GenBank (http://www.ncbi.nlm.nih.gov/) (accessed on 21 February 2021).

### 4.3. RNA Isolation and Quantitative RT-PCR Validation

Total RNA from the whole body or different tissues (head, midgut, haemolymph, fat body, epidermis) was extracted using a TRIzol reagent (Invitrogen, Carlsbad, CA, USA) according to the manufacturer’s recommendations. The concentration of RNA samples was evaluated according to the absorbance at 260 nm and the purity of RNA was confirmed by the OD260/280 ratio using a 2100 Bioanalyzer (Agilent Technologies, California, CA, USA). Then, the RNA integrity was verified by using 1% agarose gel electrophoresis. Subsequently, 1 μg of total RNA was used to synthesize the first-strand cDNA with a PrimeScript^®^ RT reagent kit (Takara, Dalian, China) following the manufacturer’s instructions. The total qRT-PCR reaction volume was 20 μL, containing 10 μL of 2 × iQTM SYBR^®^ Green I, 1 μL of 10 μM primer of each of the forward and reverse primers, 2 μL of cDNA template, and 6 μL of RNA-free enzyme water. The qRT-PCR validation was conducted using a Roche LightCycler 96 (Roche, Switzerland) and the programme was as follows: 95 °C for 5 min and 40 cycles of 95 °C for 15 s and 60 °C for 31 s. Each sample was repeated three technical times. Meanwhile, the no-template controls (NTCs) of each primer were negative with non-detection of Cq value. The *elongation factor-1 alpha* (*EF1*) (GenBank accession: DQ192234.1) and *glyceraldehyde-3-phosphate dehydrogenase* (*GAPDH*) genes (GenBank accession: MZ393966.1) were used as the reference genes to normalize the mRNA expression levels. LightCycler^®^ 96 software (Roche, Switzerland) was used to analyse the qRT-PCR results. The relative expression levels were calculated using the 2^−ΔΔCt^ method [39]. The primers were designed using the Primer-BLAST online programmer (https://www.ncbi.nlm.nih.gov/tools/primer-blast/) (accessed on 21 February 2021) (Table S1). Each sample was run in triplicate for technical repeats, and three biological replicates were performed simultaneously.

### 4.4. RNA Interference in S. litura Larvae

The oligonucleotide sequences were designed using BLOCK-iTTM RNAi Designer (https://rnaidesigner.thermofisher.com/) (accessed on 21 February 2021) (Table S2). *dsSlFAS1* and *dsSlFAS2* were synthesized in vitro using a Transcription T7 kit (Taktableara Biotechnology Co. Ltd., Dalian, China) following the manufacturer’s protocol, and the dsRNA of the green fluorescent protein (GFP) gene was synthesized as the negative control. A NanoDrop 2000 spectrophotometer was used to detect the concentration and purity of synthesized dsRNA, and 1% agarose gel electrophoresis was used to examine the RNA integrity.

*dsSlFAS1* and *dsSlFAS2* (500 ng) were injected into the third abdominal segment of 1-day-old third instar *S. litura* larvae using a microsyringe (Drummond Scientific, Broomall, PA, USA), respectively. The samples were collected 24 and 48 h after *dsGFP*- and *dsSlFAS*- were injected, and then RNA samples were extracted and the cDNA was synthesized to verify interference efficiency using qRT-PCR. These procedures were the same as described above.

### 4.5. Determination of Fatty Acid Content

The fatty acid content was determined according to the method described by Wang et al. [13] with some minor modifications. In brief, third instar *S. litura* larvae were collected 24 and 48 h after dsRNA injection, and dried at 65 °C for fatty acid determination. Each individual was weighed, and transferred to a 10 mL centrifuge tube, and then mashed with a plastic pestle to which 2 mL of n-hexane was added. The homogenate was centrifuged at 10,000 rpm for 10 min, and then 500 μL of supernatant was transferred to a new 10 mL centrifuge tube, adding 1.5 mL of n-hexane and 2 mL of 0.5 mol L^−1^ KOH-CH3OH solution. The mixture was reacted at 60 °C for 1 h. After the mixture was cooled to room temperature, 4 mL of ultrapure water was added, and the resulting mixture was centrifuged at 10,000 rpm for 10 min. The supernatant was removed to 2 mL Eppendorf (EP) tubes for gas chromatography (GC) analysis [40]. The content of methyl α-linolenate, methyl palmitate, methyl oleate, and methyl linoleate was detected using an Agilent 6820 gas chromatograph (Agilent, Santa Clara, CA, USA) equipped with a Supelco capillary column (HP-INNOWax, 30 m × 0.25 mm, i.d. = 0.20 μm; Agilent), a split injection port, and a flame ionization detector. The initial oven temperature was 200 °C, held for 1 min, then increased to 240 °C at 1.5 °C min^−1^ and held for 1 min. The detector was set at 280 °C and the injector was set at 250 °C. Nitrogen was used as the carrier gas at a flow rate of 1 mL min^−1^. The split ratio was 50:1 and the sample size was 1 μL [40].

The content of four fatty acids was calculated according to the standard curves for methyl palmitate, methyl oleate, methyl linoleic, and methyl linoleate, respectively. The content of fatty acids was measured based on content per capita (total content divided by individual weight). For each treatment, 15–20 individuals were determined.

### 4.6. Measurements of Triglyceride Content

The triglyceride content of the fat body was measured using a triglyceride ELISA kit (BYabscience, China) 24 and 48 h after dsRNA injection, following the manufacturer’s instructions. Briefly, the fat body of the larvae was collected and then homogenized in 1 × phosphate-buffered saline (PBS, PH = 7.4). The samples were centrifuged for 10 min at 12,000 rpm at 4 °C, and the supernatants were removed to new 1.5 mL EP tubes for detection. Subsequently, the supernatant was transferred into a 96-well plate, incubated with the reaction mix at room temperature in the dark for 30 min, and measured using a spectrophotometer (Thermo 1500, Waltham, MA, USA). The experiments were repeated three times.

### 4.7. Nile Red Staining

The lipid of fat bodies in third instar larvae was observed by staining with Nile red [30]. The fat bodies of *S. litura* larvae were dissected and washed with 1 × PBS (pH = 7.4) three times, each washing procedure lasting 5 min; subsequently, the fat bodies were fixed with 4% paraformaldehyde on a glass slide for 30 min at 4 °C. After fixation, the samples were washed with 1 × PBST (1 × PBS containing 0.1% Triton and 0.05% Tween20) three times for 5 min each. For lipid staining, the samples were incubated for 30 min in a 1:1000 dilution of 1 mg ml^−1^ Nile red solution (Aladdin, Shanghai, China) in 1 × PBST, and then rinsed three times with 1 × PBS (pH = 7.4). The samples were visualized using a fluorescent microscope (LSM 710, Carl Zeiss, Germany) at Ex 543/Em 626 nm.

### 4.8. Development of S. litura

To investigate the effect of SlFASs on the growth and development of *S. litura* larvae, we measured the weight and survival rate of third instar *S. litura* larvae and the emergence rate of fifth instar *S. litura* larvae after silencing the *SlFAS*, respectively. One-day-old third instar *S. litura* larvae were injected with *dsSlFAS* or *dsGFP* and weighed daily until pupation. A total of 30 third instar *S. litura* larvae were tested. To test the effects of silencing *SlFAS* on the metamorphosis of *S. litura*, the morphology and the emergence of the pupae were observed after injecting *dsSlFAS1* into fifth instar *S. litura* larvae. Each treatment was tested on 30 individuals and the *dsGFP*-injection groups were taken as the control.

### 4.9. Content of Juvenile Hormone and Ecdysone in Haemolymph of Fifth Instar S. litura after Silencing SlFAS

To determine the effects of silencing *SlFAS* on the dynamics of the juvenile hormone and ecdysone in the haemolymph of fifth instar *S. litura* larvae, the content of the juvenile hormone and ecdysone in larvae haemolymph was measured using an insect juvenile hormone and ecdysone ELISA kit (Rui Fanbioon, Shanghai, China) according to the manufacturer’s protocols. In brief, the haemolymph was collected from the fifth instar *S. litura* at 24 and 48 h after *dsSlFAS1* injection and centrifuged at 1000× *g* for 5 min at 4 °C. The supernatant was transferred into a new centrifuge tube. Subsequently, 10 μL of supernatant was added to a 96-well plate and incubated with the reaction mixture for 15 min at 37 °C. The optical density value was read at 450 nm using a spectrophotometer (Thermo1500, Waltham, MA, USA). Each sample contained three biological replicates.

### 4.10. Effect of Silencing SlFAS1 on the Expression of Other Key Genes in the Lipid Synthesis Pathway

To explore the effects of *SlFAS1* on other genes in the lipid synthesis pathway in the third instar *S. litura* larvae, the expression levels of *acetyl-CoA carboxylase* (*ACC*), *Desaturase* (*Desat*), and *Lipase*, which were key genes in the lipid synthesis pathway, were validated 24 and 48 h after *dsSlFAS1* or *dsGFP* injection by qRT-PCR; the procedures were the same as mentioned above. The primers of each target gene are listed in Table S1.

### 4.11. Statistical Analysis

Mann-Whitney tests were used to analyse the significant differences in fatty acid content; data analysis was performed using R software 3.4.0 (R Development Core Team, Vienna, Austria) [41]. One-way analysis of variance (ANOVA) followed by Tukey’s post-hoc test was used to compare the differences in the relative expression levels and the triglyceride content, as well as the content of juvenile hormone and ecdysone. 

## 5. Conclusions

In the present study, we studied the function of *FAS* genes in a major pest, *S. litura*, and found that silencing *SlFAS1* of the third instar *S. litura* larvae can depress the synthesis of fatty acids and the content of triglycerides in the fat body and, furthermore, affect development. Meanwhile, we also demonstrated that *SlFAS1* is necessary for the metamorphosis of *S. litura*, and ecdysone alteration may be involved in this process. This study provides a new insight into lipid synthesis in insects, especially in insect pests, and would provide novel targets in pest management.

## Data Availability

All data used in the work have been included in the manuscript.

## Supplementary Materials

The following supporting information can be downloaded at: https://www.mdpi.com/article/10.3390/ijms23169064/s1.
