# Supplementary material for: The Key Role of Fatty Acid Synthase in Lipid Metabolism and Metamorphic Development in a Destructive Insect Pest, Spodoptera litura (Lepidoptera: Noctuidae)
Source: Int J Mol Sci. 2022 Aug 13;23(16):9064. doi: 10.3390/ijms23169064 (PMC9409488; doi:10.3390/ijms23169064)
Supplement: Supplementary file 1 [file ijms-23-09064-s001.zip › supplementary-table.pdf]

Table S1 Primers used for qRT-PCR of lipogenesis-related genes in *Spodoptera litura*

Gene name	Primer sequence（5'–3'）	Product size(bp)
Fatty acid synthase 1 F	GTGTGGTTCGTGTTCTCC	149
Fatty acid synthase 1 R	GCCTCCGTGATGATGTTG	
Fatty acid synthase 2 F	TCCTCTACGCTCTCCTAAC	253
Fatty acid synthase 2 R	ACGATGTCCACGGTATGA	
Desaturase F	ATGTCTTTGTGGGATAGG	139
Desaturase R	CGATAAGGTCAGGGTTTG	
acetyl-CoA carboxylase F	CGCCTTCCAGAACAATGACAT	165
acetyl-CoA carboxylase R	TCTAACGCACTCTTGAACTCCT	
Lipase F	TAAGGAGATAGAGCCAGAGTTCTT	137
Lipase R	TCTTCATCATTCCGTAGTCATAGTC	
EF1 F	GAAACCGCTAAATACTATGTCA	161
EF1 R	CGGGTCTGTCCGTTCTTC	
GAPDH F	CTGCTAAAGCCGTAGGAAA	113
GAPDH R	CTGCTAAAGCCGTAGGAAA	

Table S2 Oligonucleotide templates used to synthesize siRNA

Gene Name	Primer sequence（5'–3'）
Oligo- 1 Fatty acid synthase 1	GATCACTAATACGACTCACTATAGGGCCAATACTACGGCAACAATTT
Oligo-2 Fatty acid synthase 1	AAATTGTTGCCGTAGTCTTGGCCCTATAGTGAGTCGTATTAGTGATC
Oligo-3 Fatty acid synthase 1	AACCAATACTACGGCAACAATCCCTATAGTGAGTCGTATTAGTGATC
Oligo-4 Fatty acid synthase 1	GATCACTAATACGACTCACTATAGGGATTGTTGCCGTAGTCTTGGTT
Oligo- 1 Fatty acid synthase 2	GATCACTAATACGACTCACTATAGGGGCATTGAACCTGATTACATTT
Oligo- 2 Fatty acid synthase 2	AAATGTAATCAGGTTCAATGCCCCTATAGTGAGTCGTATTAGTGATC
Oligo- 3 Fatty acid synthase 2	AAGCATTGAACCTGATTACATCCCTATAGTGAGTCGTATTAGTGATC
Oligo- 4 Fatty acid synthase 2	GATCACTAATACGACTCACTATAGGGATGTAATCAGGTTCAATGCTT
Oligo-1GFP	GATCACTAATACGACTCACTATAGGGGGGATGTCTCACATCTTGTTT
Oligo-2GFP	AAACAAGATGTGAGACATCCCCCCTATAGTGAGTCGTATTAGTGATC
Oligo-3GFP	AAGGGATGTCTCACATCTTGTCCCTATAGTGAGTCGTATTAGTGATC
Oligo-4GFP	GATCACTAATACGACTCACTATAGGGACAAGATGTGAGACATCCCTT
