# Supplementary figures and images for: The Key Role of Fatty Acid Synthase in Lipid Metabolism and Metamorphic Development in a Destructive Insect Pest, Spodoptera litura (Lepidoptera: Noctuidae)
Source: Int J Mol Sci. 2022 Aug 13;23(16):9064. doi: 10.3390/ijms23169064 (PMC9409488; doi:10.3390/ijms23169064)

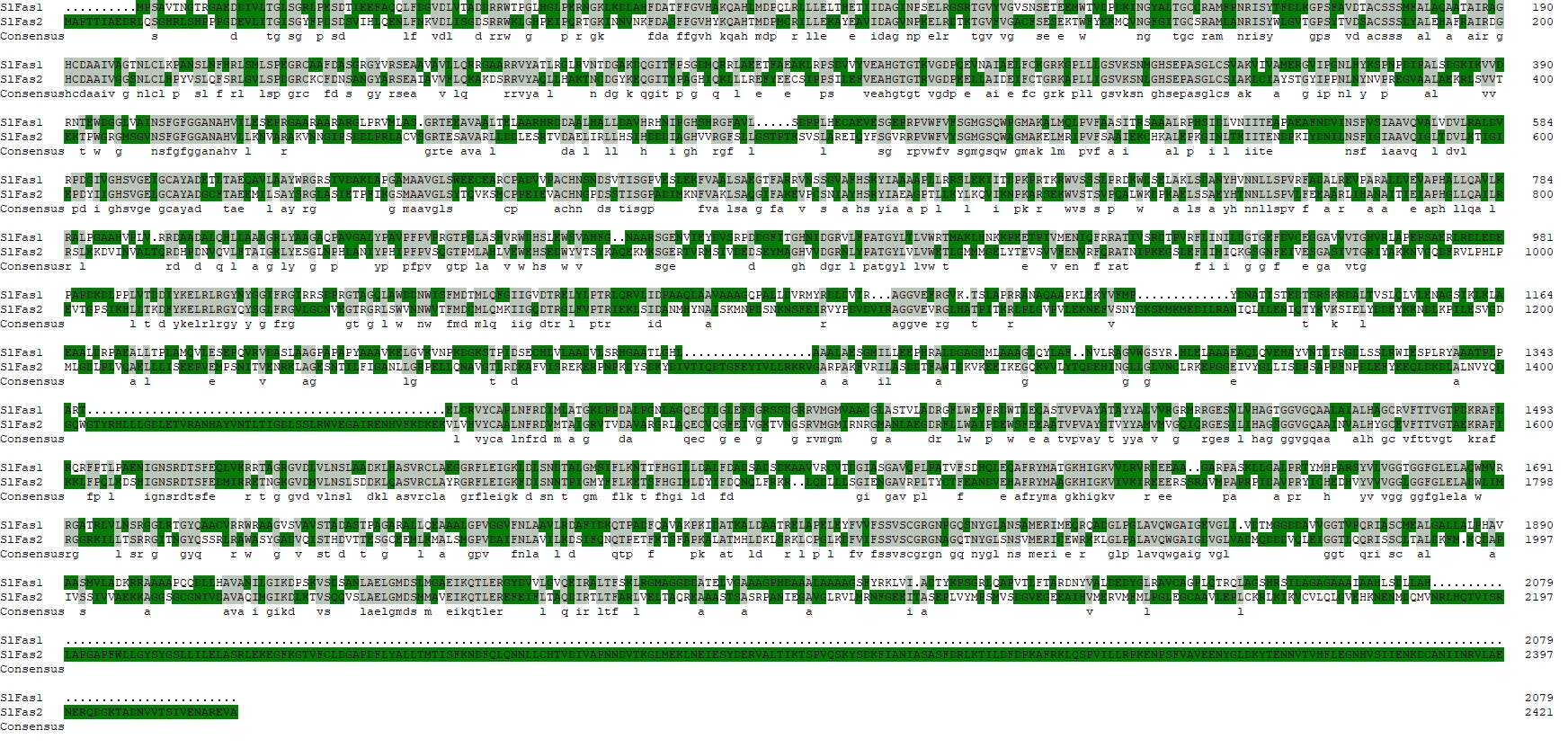

Supplement: Supplementary file 1 [file ijms-23-09064-s001.zip › Figure S1.jpg]
